# Nanopore-CMOS Interfaces for DNA Sequencing

**DOI:** 10.3390/bios6030042

**Published:** 2016-08-06

**Authors:** Sebastian Magierowski, Yiyun Huang, Chengjie Wang, Ebrahim Ghafar-Zadeh

**Affiliations:** Department of Electrical Engineering and Computer Science, York University, 6400 Keele St, Toronto, ON M3J-1P3, Canada; yiyun@eecs.yorku.ca (Y.H.); chengjie@eecs.yorku.ca (C.W.); egz@eecs.yorku.ca (E.G.-Z.)

**Keywords:** nanopore sensors, microelectronics, sequencing, CMOS, nanopore arrays, transimpedance amplification

## Abstract

DNA sequencers based on nanopore sensors present an opportunity for a significant break from the template-based incumbents of the last forty years. Key advantages ushered by nanopore technology include a simplified chemistry and the ability to interface to CMOS technology. The latter opportunity offers substantial promise for improvement in sequencing speed, size and cost. This paper reviews existing and emerging means of interfacing nanopores to CMOS technology with an emphasis on massively-arrayed structures. It presents this in the context of incumbent DNA sequencing techniques, reviews and quantifies nanopore characteristics and models and presents CMOS circuit methods for the amplification of low-current nanopore signals in such interfaces.

## 1. Introduction

In concept, nanopore sensors are a simple technology that is capable of quickly characterizing individual molecules. Essentially, nanopores are minute holes through which molecules can be snugly threaded (i.e., translocated). From an appropriately-designed nanopore apparatus, an electronic signal is generated as a consequence of this translocation; critically, this signal is indicative of the molecule’s structure and can therefore be used to decode its make-up. In theory, such sensors may be applied to any molecular complex, but nanopores have proven especially appropriate for the analysis of biological molecules, such as the nucleic acids (DNA, cDNA, RNA, etc.) where simply identifying the polymer’s residue sequence (i.e., sequencing) is indispensable for modern genomic sciences and their application. Indeed, hardware for sequencing based on this approach has already become available to researchers and shown the ability to acquire useful results in environments much broader than the traditional genomics laboratory.

Several properties of nanopore-based sensors (NBS) profoundly distinguish them from incumbent sequencing methods, including the next-generation sequencers (NGS) that dominate the industry today. This distinction has prompted some reference to NBS sequencing as a third-generation (and even fourth-generation) method. As discussed in Section 2, the properties unique to NBS have particular relevance for the fidelity of DNA measurements and their subsequent use in bioinformatics analyses. In this review, however, we also highlight the opportunities of NBS to interface with the complementary-metal-oxide-semiconductor (CMOS) technology that constitutes the vast majority of microelectronic components in computing and communications devices (i.e., information technology (IT)). The benefits of this interface apply to both the construction of the sensor itself, as well as its connection to sophisticated analogue and digital circuitry. Such a combination bodes very well for the future of NBS; tied closely to CMOS, it promises a molecular sensing technology that not only benefits from direct access to sophisticated electronic signal processing and computation, but also the opportunity to benefit from the computing industry’s scaling trajectory as exemplified by Moore’s law.

In this paper, we review the state-of-the-art NBS/CMOS interfaces with an emphasis on the arrayed sensor methods of most interest to DNA sequencing applications. We also take the opportunity to present in more detail the context in which NBS as a sequencing method arose by briefly reviewing in Section 2 sequencing technologies in general, the current state-of-the-art and how NGS has already incorporated CMOS interfaces itself. In Section 3, we review the physical construction of biological and solid-state nanopores with a detailed consideration of their key characteristics; properties that can be significantly influenced by the interface design. Section 4 discusses the manner in which state-of-the-art interfaces between nanopores and CMOS have been realized for both biological and solid-state sensor instantiations. In Section 5, key issues and considerations for CMOS amplification at its interface to NBS is reviewed and discussed. Section 6 concludes the paper.

## 2. Nanopore Sequencing in Context

Before detailing nanopore technology and its interface to CMOS technology for the purpose of sequencing, it is worthwhile to outline existing sequencing technologies, their abilities and limitations and, thus, establish the context in which nanopore/CMOS advances are being made, as well as the degree to which they compare to established performance metrics.

### 2.1. Sanger and Next Generation Sequencing

Fred Sanger’s DNA sequencing technique [1], a means employing randomly-interrupted enzymatic extension, was a profound contribution to genomic science. In short, this technique employs the DNA polymerase (DNAP) to synthesize double-stranded (ds) DNA from a (sufficiently primed) single-stranded (ss) DNA isolate (the template). Among the mixture of standard deoxyribonucleoside triphosphate (dNTPs) substrates, adenine (A), cytosine (C), guanine (G) and thymine (T), used by DNAP to synthesize the dsDNA, are also included the chemically-altered di-dNTP (ddNTP) elements that are capable of arresting DNAP’s extension mechanism. That is, once a ddNTP is attached to the template, DNAP is prevented from adding more dNTPs (or ddNTPs). With identical copies of ssDNA mixed among the dNTPs and ddNTPs, the outcome of this reaction is a set of dsDNA of various lengths whose terminating nucleotides can be identified and thus sequenced by the resulting molecule size. Sanger’s contribution accelerated the process of DNA sequencing from rates of roughly 10 base pairs (bp) per year to about 100 bp/day [2]. The Sanger method, with refinements, remained the dominant means for sequencing DNA until a decade ago, and its low-cost justifies its presence in may labs to this day. Among two outstanding improvements worth of note was the adoption of fluorescent labelling [3] with its associated optical detection hardware in the mid-1980s. In this advance, nucleotides are bonded to fluorescent molecules (fluors) capable of emitting a light unique to the dNTP with which they share a bond. The ability to shine a light and register an identifying reflection colour greatly helped the automation of the sequencing process. The introduction of capillary transport in place of gel electrophoresis was another key advance that itself was enabled by the introduction of fluorescent labelling. The capillary technology increased sequencing throughput to 360 kilobase pairs (kbp) per day around the time of its introduction in the late 1990s [4] and through refinements is today capable of processing roughly 1–2 million bp/day.

**Figure 1:**
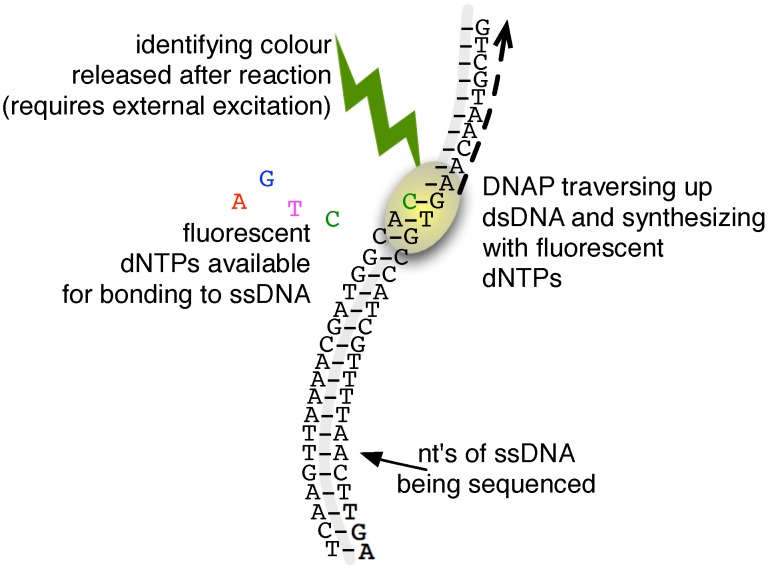
A representation of the sequencing-by-synthesis procedure. A dsDNA is synthesized base-by-base. Each reaction can be identified with a unique signal, and hence, the strand’s sequence can be identified.

A substantial advance in DNA sequencing methods started in 2005 with the emergence of a variety of so-called next-generation-sequencing (NGS) techniques. Where the Sanger-based methods effectively sequence by attaching a single detectable base to a random location in an analyte molecule (a process repeated and aggregated over a multitude of samples to eventually arrive at a coherent sequence), the NGS machines achieve sequencing by adhering, to a single-stranded DNA, multiple detectable bases in a controlled and sequential process (and thus, synthesizing a double-stranded chain), a method generally referred to as sequencing-by-synthesis; an abstract illustration of this approach is shown in Figure 1. This approach makes it possible to carry out the sequencing in situ without the need for the physical transport of the analyte as needed in the Sanger method. As a result, NGS has opened and exploited the possibility of realizing much more complex and compact sequencing platforms. This, in turn, has lead to the construction of multi-channel systems and, thus, achieved high-throughput operation with institutional NGS machines capable of exceeding 1 Tbp/day and even some desktop NGS machines operating over 100 Mbp/h [5].

Important examples of NGS technologies employing the sequencing-by-synthesis strategy include the Solexa/Illumina bridge amplification method, which dominates the field today [6], as well as Roche’s pyrosequencing method [7] with bead-emulsified polymerase chain reaction (PCR) [8] and Ion Torrent’s (now Thermo Fisher) pH sensing technique [9]. A feature common to all of these platforms, as well as their Sanger-based predecessors, is the need for clonal amplification of the DNA under study via some form of PCR [10]. In other words, to achieve sequencing with sufficient fidelity, multiple copies (roughly one million) of analyte molecules must be made; the reason for this is the limited sensitivity of detection methods in NGS (a topic we return to shortly). Although each NGS platform employs ingenious methods to implement its PCR at ever more impressive scales (in terms of individual size and aggregate number of reaction centres) and performance levels, this step remains a critical impediment to visions of ubiquitous, low-cost sequencing platforms given the relatively sophisticated chemical preparation and control it requires. Arguably, an even more immediate drawback to the reliance of NGS systems on clonal amplification is the compromised diagnostic ability of the sequencer. This stems simply from the errors incurred during the amplification procedure’s copy process and its inability to retain molecular variants, such as methylation, a critical marker for the study of epigenetic phenomena. The ability of nanopore methods to sequence without resorting to chemical amplification has the potential to avoid this problem [11,12,13].

Another limitation of NGS systems is their relatively short read-length, that is the number of nucleotides (nt) that can be sequenced using NGS processes in a single run to a given accuracy. The availability of only short reads naturally leads to difficulties in the de novo extraction of significant long-range patterns (e.g., genes) from the DNA under investigation. This problem is especially acute for complex genomes and in such cases, NGS data require the availability of an existing reference sequence to which its measurements can be aligned [14].

Leading NGS machines achieve read-lengths of roughly 100–200 nt with refinements managing to reach lengths of 300 in some cases [15]. By contrast, typical runs of Sanger-based sequencing obtain adequately accurate reads in the range of 1000 nt [16]. As with the cloning step, chemistry is the cause underlying this NGS read-length limitation. In particular, the ideally methodical process of sample molecule extension characteristic of the sequencing-by-synthesis approach (as illustrated in Figure 1) is compromised by intermittent bonding errors (e.g., molecules are extended by more than one nt, or not at all, or incompletely in the case of homopolymer chains) and incorrect or residual fluorescence signals. As a result, the more chemical reactions to which a DNA molecule under sequence is subject, the more errors it accumulates. This accumulation effectively lowers the signal-to-noise ratio available to detectors from long reads and, thus, limits NGS to the run lengths noted above.

### 2.2. Next Generation Sequencing CMOS Interfaces

All NGS platforms ultimately leverage CMOS technology in the form of high-speed computing elements (i.e., traditional microprocessors and their customizations, such as graphical processor units) to convert their raw measurements into actual DNA sequence predictions. A far more intimate example of this interface, however, is the charge-sensing machine first deployed in the market by Ion Torrent (IT) [17].

Rather than relying on the optical detection of tagged nucleotides, the IT process, also a sequencing-by-synthesis approach, hinges on detecting the charge by-products of reactions during extension. Most importantly, this charge can be sensed with the ion-sensitive-field-effect-transistor (ISFET), a device long known for its ability to detect ambient charge [18,19], but only recently scaled to unprecedented levels of density by IT in a commodity-grade CMOS technology coupled with unobtrusive low-temperature post-processing steps.

**Figure 2:**
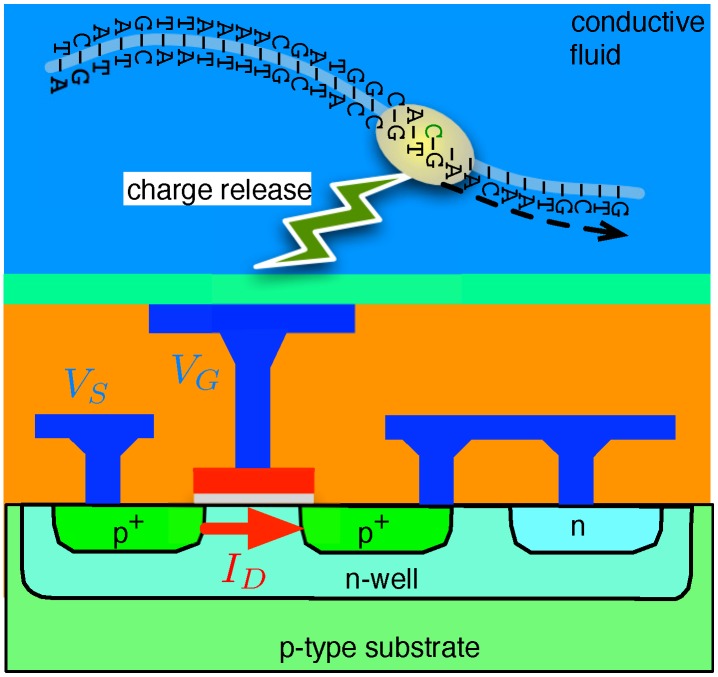
Sequencing-by-synthesis as achieved with ion-sensitive-field-effect-transistor (ISFET) charge sensors.

A simplified example of this IT’s charge-sensing apparatus is shown in Figure 2. As portrayed, a p-channel metal-oxide-semiconductor (PMOS) transistor immersed in a bath of conductive fluid is used to conduct a given current $I_{D}$ under a given gate bias, $V_{G}$. The reaction incurred due to the binding between a single-sided strand of DNA and a complementary nucleotide releases positive charges (hydrogen ions) that bind to a sensitive area above the PMOS and thus vary its threshold voltage *V*-th. As a result, the transistor’s source voltage $V_{S}$ is changed in relation to the released charge, thus achieving a pH sensor.

Each ISFET in the IT method outlined above can be viewed as the primary constituent of a pixel in a charge-detecting imager. This is in direct analogue to optical imagers, such as those based on charge-coupled-device (CCD) pixels commonly employed in sequencing systems dependent on fluor labels.

However, IT’s reliance on an alternate signal modality (i.e., charges vs. photons) has allowed it to achieve much smaller sensor dimensions and, hence, much smaller pixel sizes, than their optical counterparts. Furthermore, the ability to interface charge sensors directly to the miniature chemical reactors in which the chemical sequencing reactions take place further distinguishes IT charge sensors from their optical counterparts, which often employ sophisticated fibre-optic links between the chemistry and detection modules.

As a result of the advantages noted above, the IT technology has demonstrated the significant benefits of interfacing CMOS technology to sensors for sequencing; for example, in 2012, in a mature 110-nm CMOS technology (22-nm FinFETCMOS technology had just entered full-scale production at the time), over 150 million ISFET pixels were realized in one chip (the PI) roughly 20 cm on a side [15]. Given that the first in-CMOS ISFET demonstration only occurred in 1999 [20] and the first ISFET CMOS array to be described integrated only four devices [21,22] underscores the swiftness of IT’s development and the potential for CMOS bio-interfaces. Nonetheless, as noted earlier, this approach, given the accumulation of noise during its sequencing process, is still subject to arrested read lengths.

### 2.3. Single-Molecule Sequencing

Arguably, the next major step in the evolution of sequencing machines into their third generation centres around the creation of single-molecule detection technologies. Such a development promises to address two critical complications in NGS machines: chemical amplification and short read-lengths. The former issue is eliminated practically by definition, a scheme capable of detecting the characteristics of single molecules obviously foregoes the need to operate on multiple copies of that molecule; the latter deficiency is essentially mitigated by the introduction of a short-memory (ideally zero-memory) detection scheme.

Nanopore-based sequencers are one embodiment of this vision, and we detail them and their interface to CMOS technologies below. In the spirit of establishing a broader context, however, we first outline its contemporary on the single-molecule sequencing front, Pacific Bioscience’s (PacBio) focused optical detection system [23]. In this technology, a nanoscale well (100-nm in diameter) with a special coating has a modified DNAP adhered to its bottom. The minuscule well structure achieves accurate focusing of light, a zero-mode waveguide (ZMW), capable of directing light originating from close proximity to the DNAP to an optical detection system [24].

**Figure 3:**
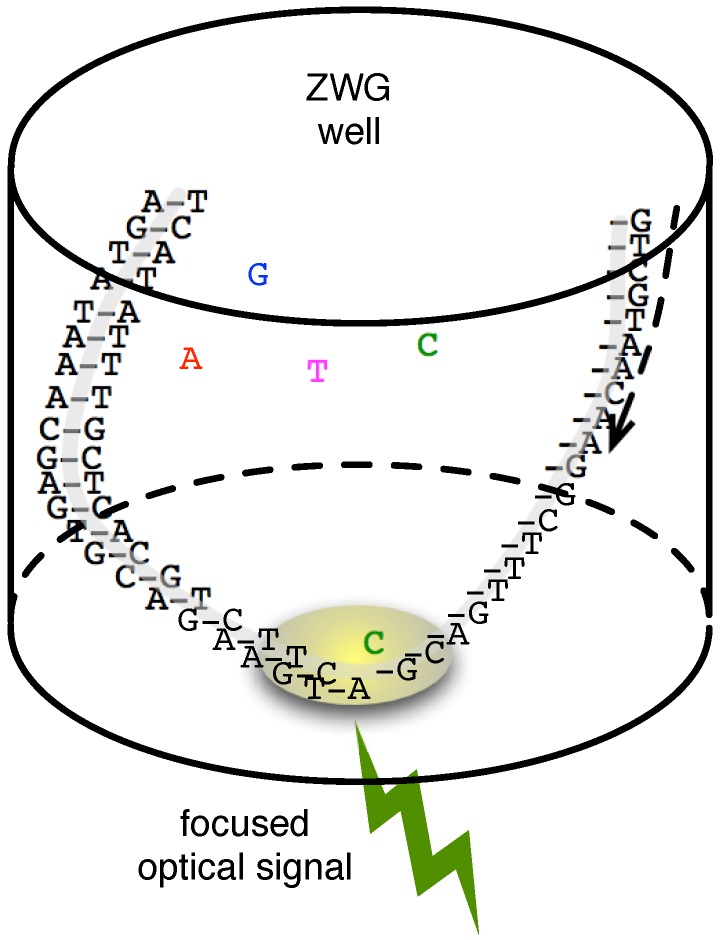
Strand sequencing confined to a signal focusing zero-mode wave waveguide as described in [14].

As with optical NGS schemes, the PacBio system has its DNAP attach fluor-tagged nucleotides to a ssDNA template (see Figure 3). Because of the ZMW, however, such an incorporation can be sensed, in real-time, by exciting the ZMW with a laser and recording the colour of the reflected light. A stimulated emission signal is sensed as soon as a labelled dNTP is sampled by the DNAP and, for matching samples, continues during the process of incorporation onto the template. Since the fluor tags are linked to the terminal phosphate moiety, the reflected signal ceases and, therefore, terminates the single-molecule signature, once the DNAP cleaves the phosphate group (causing the fluor to diffuse out of the focus region), a by-product of its extension mechanism.

PacBio’s detection method, unencumbered by accumulated signal degradation, has allowed it to achieve read-lengths in excess of 10-kbp at rates of roughly 1–5 bp/s/well. The minute ZMW footprint allows the realization of a massively parallel system (over 100,000 have been achieved [14]) with high throughput. An early demonstration of the system achieved roughly 85% accuracy per read with the ability to exceed 99% over a 15-fold consensus. To control sequencing accuracy, not only must the ZMW and the optical detector be sufficiently engineered, but so does the DNAP itself. One goal is to ensure that the enzyme’s dNTP incorporation process is made slow enough and, hence, its optical pulse (i.e., its single-molecule signature) made to achieve sufficient temporal duration to distinguish it from error events (e.g., background emission, incompatible dNTP sampling, etc.). In one example, this has resulted in legitimate signal pulse durations of 100-ms (on average) relative to non-idealities in the range of 10 s of μs to sub-millisecond [23]. Temporal spacing (on the order of 200 ms) between pulses is a related feature dependent on the engineering of the DNAP’s translocation and dNTP binding times.

While PacBio’s single-molecule method presents a significant breakthrough compared to incumbent technologies, its reliance on a synthesis-based optical signalling technique aligns it closely with the Sanger and NGS lineage. Although, this certainly does not preclude the interface of PacBio’s system to CMOS (and all its attendant computational, economical and scaling advantages), it certainly makes such a merger very awkward; as a consequence, largely, of the relative inferiority of CMOS to serve all aspect of a stimulated emission procedure.

Nanopore-based sequencing breaks both of the key features (synthesis and optics) established by its predecessors: (i) its signalling process works directly on the DNA isolate without any need for a dNTP-by-dNTP construction and the chemical complexities this entails (e.g., nucleotide modification, amplification, nucleotide addition, wash, fluor removal, blockers, etc.); (ii) it associates structural DNA features with electronic charge rather than photonic wavelength making an interface to production-grade CMOS much more amenable. We now detail the function, the features and future directions of this method.

## 3. Nanopores

Nanopore-based sequencers (NBS) are a very recent addition to the sequencing tool marketplace. Most prominently, a nanopore-based molecular sensing device plus accompanying sequence analysis tool chain have been made available by Oxford Nanopore Technologies Inc. (ONT) since the spring of 2014 [25,26]. These systems currently achieve sequencing speeds on the order of 100 nt/s, orders of magnitude faster than the methods outlined above, with the potential for vastly higher per-sensor speeds given a quality interface to a sufficient signal processing technology.

**Figure 4:**
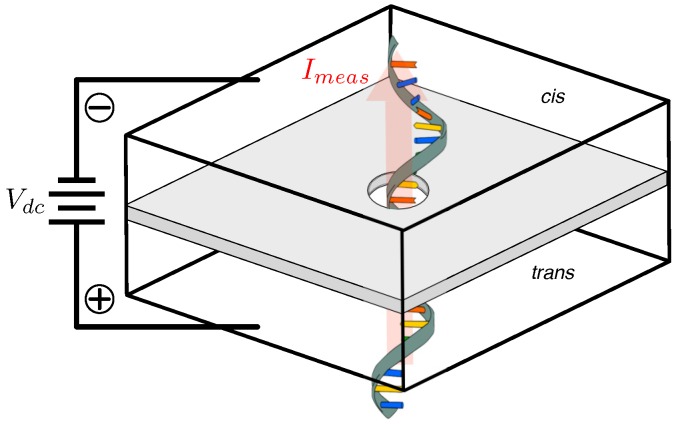
Oblique view of simplified nanopore structure. A thin, pore-infused, membrane separates the *cis* and *trans* chambers biased with DC voltage $V_{dc}$.

Despite its nascent presence in the market, the core functional idea behind NBS parallels the Coulter particle counter [27] of the late-1940s and, thus, at least conceptually, pre-dates even Sanger-sequencing as a means of nucleic acid analysis for the bio-sciences. Coulter detection works by sensing the analyte-dependent variation in the conductivity of an opening or channel separating two chambers (the *cis* and *trans*) infused with conductive fluid (e.g., the salts NaCl or KCl dissolved in water). An idealized arrangement of this mechanism is illustrated in Figure 4.

**Figure 5:**
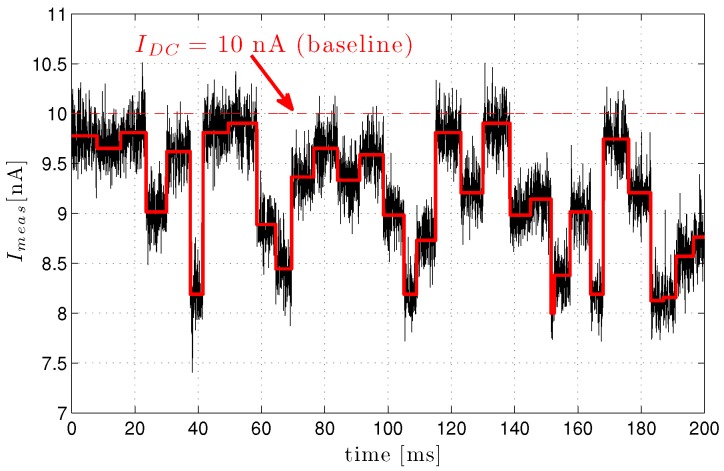
Example illustration of modulated current through a nanopore.

Applying a potential across this apparatus causes the fluid’s ions to flow through the hole from one channel to the other, thus establishing a DC baseline current, $I_{dc}$. As illustrated in Figure 5, introducing analyte particles into say the *cis* bath and allowing them to traverse (i.e., translocate through) the hole by any of a number of mechanisms (e.g., electrostatic, pressure/concentration gradient, mechanical, etc.) results in intermittent blockage of the opening, a resultant drop in conductivity and, hence, a modulation of the baseline current whenever a particle translocates the opening. Ideally, a piecewise-constant measured current response as outlined in red in Figure 5 may be anticipated; this is a result of discrete molecular features imposing characteristic blockage. At the very least however, the noise of the test apparatus (black curve in Figure 5) can be expected to corrupt this, resulting in a stochastic measured current, $I_{meas}$, the handling of which imposes a significant signal processing challenge.

Of course, the more immediate problem to appropriating the Coulter method for the quantification of DNA is to find a means of shrinking its critical feature, the hole through which comparably-sized DNA analytes are filtered (the eponymous nanopore) is needed. Namely, without a sufficient match between particle size and channel diameter, the realizable $I_{dc}$ modulations are too weak to be accurately detected due to the aforementioned noise issue. In the mid-1990s, a means of achieving a workable nanosensor was forwarded, propelled by the insight that naturally-occurring protein orifices (porins) could suffice [28,29,30]. Infusing such biological nanopore structures in a lipid bilayer membrane to serve as a pore support and as a boundary between *cis* and *trans* has been common in published reports as reviewed in [31,32,33].

### 3.1. Biological Nanopores

**Figure 6:**
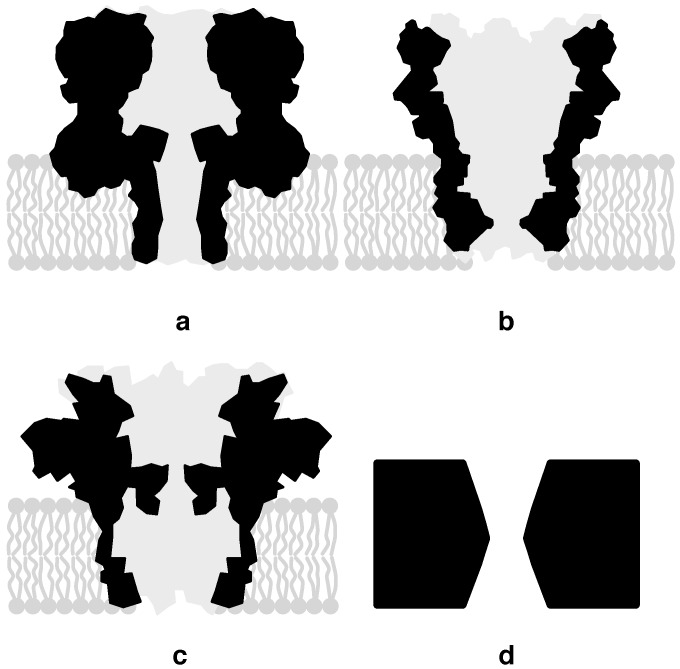
Cross-section of (**a**) the α-HL nanopore infused in a lipid bilayer membrane support structure, (**b**) MspA nanopore, (**c**) CsgG nanopore and (**d**) solid-state nanopore.

Workers quickly identified the bacterial protein alpha-hemolysin (α-HL) as a potential candidate for DNA sequencing. In nature, this is a virulence factor capable of infusing itself in the cell membrane, thus allowing uncontrolled transport of particles until lysis. This mushroom-shaped complex (see Figure 6a) exhibits a narrowed channel that inserts into a support membrane. The *β*-barrel possesses a minimum diameter of 1.4 nm and a length of 5-nm [34], a good cross-sectional fit for ssDNA (diameter 1.2 nm), but unfortunately long enough to confine about 7 nts (nt-to-nt spacing 0.7 nm in single-stranded DNA) at a time; this compromises α-HL’s ability to discern individual DNA monomer residues. Nonetheless, in 1996, Kasianowicz et al. first demonstrated the ability of ssDNA to modulate ionic currents through α-HL in relation to the molecule’s make-up (but not to the scale of individual nt constituents). Roughly, baseline currents on the order of $I_{dc}=100$ pA are observed for these pores, a value dependent on a variety of settings, including applied voltage (100–200-mV is typical for biological pores to prevent damage to the pore’s membrane support) and salt concentration (∼1 M is typically used). The modulation depth (i.e., the deviation from the baseline) reaches values ranging between 0.3 and 0.5 as a fraction of $I_{dc}$ [35].

Over the ensuing years the biological nanopore community has been very active in engineering pores with more selective channel structures, as well as additional enzymes capable of regulating the progress of DNA through the pores. For instance, *Mycobacterium smegmatis* porin A (MspA) [36] (Figure 6b) has emerged as a popular alternative to α-HL due mainly to its smaller opening (∼1.2 nm) and, more importantly, small effective thickness (∼0.6 nm), resulting in higher monomer resolution. This improvement was accompanied by a translocation rate roughly 10× greater than that exhibited by α-HL [37]. Another recent addition to nanopore sequencing systems is the CsgG protein (from the *curli specific gene* G) shown in Figure 6c, which naturally exhibits a minimum opening diameter of 9 Å [38].

From a pure information-processing perspective, high-speed DNA translocation through the nanopore is desired. However, the poor nature of the interface between both biological and solid-state nanopores and the electronics used to process their signal has resisted efforts to operate the nanopore sensor at translocation speeds natural to them. In essence, ancillary electronic components in a typical measurement apparatus combined to severely degrade high-frequency signal levels, making them indistinguishable from noise. Given the relative dominance of researchers with biochemical expertise in the nanopore field, a significant effort has been made to engineer pores capable of processing DNA samples more slowly. A popular means of doing this today is through the use of motor proteins, such and the DNA polymerase phi29 attached to the porin molecules that effectively ratchet a molecule through the pore [39,40,41]. These mechanisms have succeeded in realizing translocation rates below 100 bases/s (nt/s) far below the 1 Mnt/s that occur without any slowing mechanism.

### 3.2. Solid-State Nanopores

With regards to DNA sequencing, engineered biological nanopores (BNs) have been the first to achieve commercial success. Naturally, improved BNs are an important reason behind this, but just as vital has been the interface of this technology to CMOS (discussed in Section 4). Given their construction from the same semiconductor materials used in CMOS, solid-state nanopores (SSNs) (artificially created openings in a crystalline membrane (e.g., see Figure 6d)) hold even greater promise for such a fusion of technologies; being constructed from an inorganic material, they can also sustain much higher voltage levels leading to the availability of larger signal currents (>1 nA).

Further, SSNs, being amenable to more controlled fabrication techniques offer the possibility of wider sensing modalities. That is, besides the ionic current approach outlined above, researchers have investigated transverse electronic tunnelling (TET) [42] and capacitive sensing among others [43]. Generally speaking, these alternative approaches attempt to measure disturbances in signals perpendicular to the flow of the DNA analyte as opposed to the ionic current approach, which essentially notes disturbances in dynamical phenomena occurring in parallel with the translocation of DNA through the pore. This orthogonal-disturbance property theoretically endows these alternative methods with the potential for greater signal resolution than their blockade current-sensing counterpart.

In TET [44], nanoelectrodes separated by the SSN are realized [45,46,47] and biased. When DNA components traverse the nanopore-sized gap between electrodes, a measurable (∼1 nA) electron tunnelling current is supported, thus providing a means of identifying the molecule traversing the hole.

The capacitive sensing approach imagines a traditional planar capacitor (i.e., an insulator sandwiched between conductors) with a nanopore perforation [48,49]. As DNA bases pass the insulator, they modulate the charge on the capacitor and, hence, the voltage measured across its terminals. Although simulations indicate the potential for accurate high throughput operation, compelling experimental results remain to be demonstrated [50,51].

Since the first demonstration of SSN-based DNA sensing in 2001 [52], numerous improvements have followed, mostly focused on finer and more controllable pore construction, as well as the integration of alternate materials to augment performance, such as graphene [53]. Outstanding results in this area include an SSN with an effective thickness of 1.7 nm and a diameter of 1.4 nm [54].

**Figure 7:**
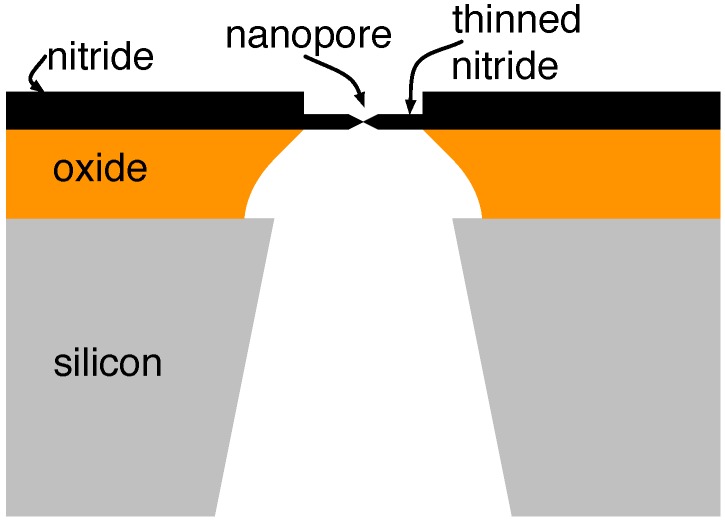
Cross-section of a typical solid-state nanopore and its support structure.

A cross-section of the resulting overall structure in this case is drawn in Figure 7 and consists of a 500-μm thick silicon, a 5-μm thick silicon dioxide and a 50-nm silicon nitride layer. Wet etch achieves the back-side silicon and oxide openings shown in Figure 7, resulting in a suspended nitride 10–20 μm in extent. A membrane-thinning plasma exposure reduces the nitride over a window of roughly 0.2×0.2 ${\mu m}^{2}$ to a thickness of roughly 5-nm, thus achieving a structure in which a small hole can be made. This allowed the application of transmission electron microscope to drill holes of the aforementioned dimensions in the thinned membrane. Similar approaches have been used to consistently achieve nanopores in amorphous silicon with a thickness below 2 nm [55].

### 3.3. Nanopore Characteristics and Models

**Figure 8:**
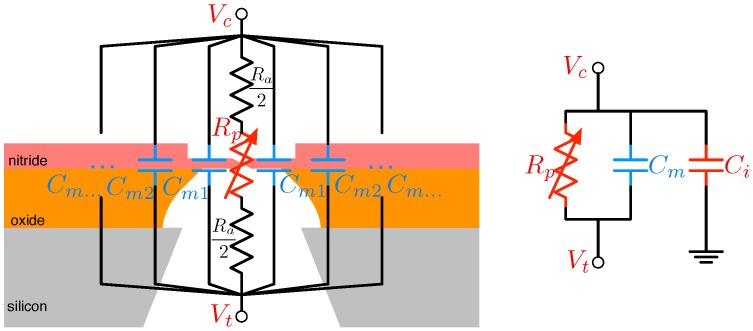
Electric circuit mode of the nanopore with applied voltages $V_{c}$ and $V_{t}$ at the *cis* and *trans* terminals.

As already mentioned, a key distinguishing characteristic of nanopore-based sequencing is its use of electrochemical current as the signal into which the physical constituents of a DNA molecule are transduced. To optimally process this current signal with a microelectronic technology, such as CMOS, a suitable electrical model is needed to guide its design. An example of such a model is shown in Figure 8.

The model’s most important component is the pore resistance $R_{p}$, a measure of the degree to which mobile charges are constricted when traversing the pore. This value changes in proportion to the molecular structure traversing the hole alongside the solution’s ions and, hence, serves as the model’s functional core.

Equivalently, the pore can be represented by its conductance $G_{p}=1/R_{p}$, which can be further imagined in terms of the difference:

(1) $$G_{p}=G_{dc}-\Delta G_{x}$$

where $G_{dc}$ is the pore conductance when only the ionic components of the electrolyte solution are traversing it under the influence of a steady DC potential. The DC potential across the pore is denoted with $V_{dc}$, and $\Delta G_{x}$ in Equation (1) refers to the conductance variation caused by the translocation of some molecular “substructure”, “feature” or “event”, *x*.

The current flowing through the conductor can similarly be described with the relation:

(2) $$I_{meas}=I_{dc}-\Delta I_{x}$$

where Ohm’s law relates the model variables with:

(3) $$I_{meas}=G_{p}\cdot V_{dc}$$

The extent to which a translocating feature makes the DC current stray (or “modulate”) is represented by $\Delta I_{x}$. From the expressions above, this is:

(4) $$\Delta I_{x}=V_{dc}\Delta G_{x}$$

indicating the possibility of a larger $I_{meas}$ should $V_{dc}$ and $\Delta G_{x}$ be increased.

Remaining resistive contributors, such as the resistance of the fluid, the spreading resistance around the pore and the electrode resistance, are lumped into the access resistance term $R_{a}$. Clearly, this is a parasitic term that attenuates the achievable $\Delta I_{x}$ for a given $V_{dc}$. In standard (high-voltage, high ionic conductivity) nanopore scenarios, the access resistance impact has not been noted as significant (e.g., ∼10 kΩ [56]), and we ignore it for the remainder of this discussion. However, under lower voltage and slower translocation rate scenarios of the type that may be important for future solid-state nanopore applications, a noticeable influence from the access region has been noted [57,58].

The membrane capacitance, $C_{m}$, is another critical element of the nanopore model. Its admittance increases as the signal dynamics at its terminals speed-up. As a result, at the frequency of $I_{meas}$ (or its spectral content) increases, the proportion of the signal still proportional to the conductive modulations $\Delta G_{x}$ drops. Other parts of a nanopore measurement apparatus impose like problems with the addition of capacitive parasitics. These are lumped into the component $C_{i}$ in Figure 8 and include contributors, such as wiring and circuit capacitance. Efficient pore and CMOS implementations succeed in substantially reducing these.

#### 3.3.1. Nanopore Conductance

For a more quantitative appreciation of the state of nanopore development, particularly SSNs, we consider the solid-state nanopore conductance model discussed in [59,60,61], where the pore’s unobstructed DC conductance is:

(5) $$G_{dc}=\sigma /\left(\frac{4t}{\pi d^{2}}+\frac{1}{d}\right)$$

in which *σ* is the electrolyte conductivity, *d* is its diameter and *t* is its thickness.

The fluctuation in the pore conductance due to the translocation of nucleic acids is also considered in [59]:

(6) $$\Delta G=G_{dc}-\sigma /\left(\frac{4t}{\pi (d^{2}-d_{na}^{2})}+\frac{1}{\sqrt{d^{2}-d_{na}^{2}}}\right)$$

where $d_{na}$ is the effective diameter of the nucleic acid traversing through the pore.

For an advanced SSN as presented in [54] (a pore with a 1.5-nm diameter, thinned nitride thickness of 5-nm and a bias of $V_{dc}=1$ V), modulation current values of $\Delta I_{x}=\left\{4.2,4.8,5.1,5.9\right\}$ nA are obtained for x= C, T, A and G, respectively. This results in a minimum separation between modulated currents of $\min\left(\left\{\Delta I_{x}\right\}-\left\{\Delta I_{x}\right\}\right)=i_{\delta ,min}=300$ pA (i.e., between the modulated currents corresponding to T and A). This separation effectively defines the signal, $i_{\delta }$ (and its strength), and is significantly higher than currently-reported BN values. Another useful signal metric is the full-scale modulation depth $\Delta I=\max(\left\{\Delta I_{x}\right\}-\left\{\Delta I_{x}\right\})$ a value that, again, can reach substantially larger quantities in SSNs than BNs.

The terms above are critical in determining the design of the CMOS interface. Clearly, $\Delta G_{x}$ influences the size of the signal that our CMOS interface needs to process. Given their common origin (i.e., as attested by a comparison of Equations (5) and (6)), $G_{dc}$ is also an indication of the achievable signal strength, with higher values of $G_{dc}$ implying the availability of larger signal currents.

**Figure 9:**
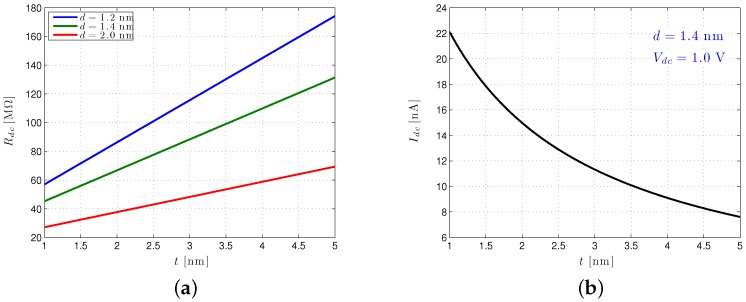
Key solid-state nanopore DC values ($R_{dc}$ and $I_{dc}$) as a function of pore thickness (*t*). (**a**) The $R_{dc}$ for a range of solid-state nanopore dimensions; (**b**) the $I_{dc}$ for a range of solid-state nanopore thicknesses.

For BNs, it is not uncommon to operate with $R_{dc}$ = 1 GΩ. Modern SSNs can achieve substantially smaller values, as illustrated in Figure 9a.

For the solid-state nanopore dimensions considered here, the effective pore resistance is quite manageable (i.e., well below 1 GΩ) for pore sizes aimed at discerning DNA phenomena. For thicknesses below 2.5 nm $R_{dc}$, greater than 100 MΩ is not expected, and for thin pores around 1.5 nm [55] intended to process the ssDNA (i.e., 1<d<2 nm), interface designers might be dealing with $R_{dc}$ as low as about 50 MΩ.

**Figure 10:**
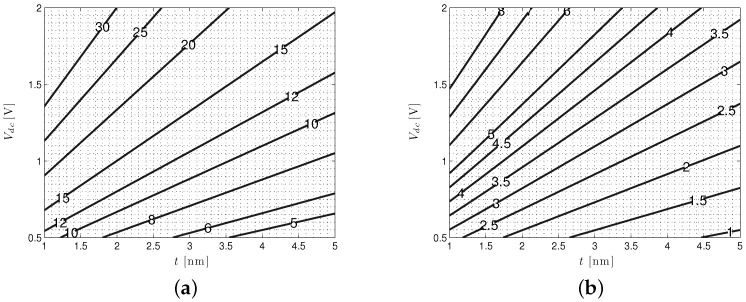
Solid-state nanopore (SSN) (d=1.4 nm) $I_{dc}$ and ΔI as a function of pore bias ($V_{dc}$) and thickness (*t*). (**a**) Contour plot of the $I_{dc}$ (in nA) as a function of nanopore thickness, *t*, and applied DC bias, $V_{dc}$; (**b**) contour plot of the ΔI (in nA) as a function of nanopore thickness, *t*, and applied DC bias, $V_{dc}$.

A plot of the $I_{dc}$ as a function of *t* is shown in Figure 9b. For the diameter and thickness indicated, it is clear that quite a high quiescent current (≈18 nA) can be achieved. Increases in *t* and *d* naturally reduce $I_{dc}$ due to the drop in $R_{dc}$ that such changes impose. A contour plot of $I_{dc}$ as a function of $V_{dc}$ and *t* is shown in Figure 10a. This presents a broader picture of the DC current range possible as the voltage across the pore is varied under different thicknesses. As already indicated, such a broad variation in $V_{dc}$ is not available to BNs, which are typically biased at settings less than 200 mV to prevent them from being damaged.

A similar approach can be used to quantify our modulated currents ΔI and their effective signal values $i_{\delta }$. In analogue to Figure 10a, a contour plot of ΔI achievable under different SSN biases and membrane thicknesses (again for d=1.4 nm) is shown in Figure 10b. As with $I_{dc}$, for state-of-the-art solid-state pores (t≈1.5 nm, d≈1.4 nm), rather high ΔI can be achieved. With a 1-V $V_{dc}$, we can approach values as high as ΔI=4.5 nA, a nice improvement over the ΔI=1.7 nA for the d=1.4 nm, t=5 nm pore described above [54]. A ΔI of 4.5 nA distributed over just four nucleotide levels gives us an average of $i_{\delta ,avg}=1.5$ nA, an extremely high value compared to the present state of the art and at least some indication of the extent to which SSN performance may yet improve.

#### 3.3.2. Nanopore Capacitance

The insulating support structure (i.e., the membrane) in which the nanopore is drilled contributes a capacitance $C_{m}$, the membrane capacitance, to the sensor’s equivalent circuit, as shown in Figure 8 for the SSN example. As indicated in the picture, this can be contributed by a variety of layers and regions each symbolized with some effective contributor $C_{mk}$ where:

(7) $$C_{m}=\sum_{k}C_{mk}$$

In the BN, the primary contributor to $C_{m}$ is the thin layer bilayer support structure, which possesses a capacitance of roughly 4.5 fF/${\mu m}^{2}$ [62]. The relevance of this number will be discussed in Section 5, but as the reader may anticipate, it is a value we seek to minimize given that the time-constant of nanopore signals will be governed by the product $R_{dc}C_{m}$; given the relatively high value of $R_{dc}$ discussed above, it is clearly incumbent on the sensor and interface designer to minimize $C_{m}$ in order to maximize the high-frequency handling ability of the sensor.

The more intricate structure of SSNs provides for more capacitive contributors [63], but arguably the most important of these is the portion of nitride material exposed to the chemical bath on both sides. This layer, roughly 50-nm thick, possesses a capacitance of roughly 1.2 fF/${\mu m}^{2}$ [63], smaller than the 10 fF/${\mu m}^{2}$ of the thinned nitride layer, but typically distributed over a much larger area.

Alongside the pore’s own membrane capacitance, ancillary contributions (e.g., wiring, package, circuit, etc.) due to the details of the connection between the sensor and its electronics give rise to the interface capacitance, $C_{i}$. In work relying on standard nanopore/electronics interfaces (discussed in Section 4), this value could easily reach values of 20 pF [64], a far cry from the roughly 1-pF setting achievable with more intimate interfacing technology.

In general, then, the design of the nanopore-CMOS interface seeks to achieve connections with minimum total capacitance:

(8) $$C_{t}=C_{m}+C_{i}$$

Minimizing this value can have profound effects on the utility of nanopore-based sequencing devices. As detailed in Section 5, this capacitance effectively reduces the impedance experienced by the measured signal as it enters the first CMOS amplification element. This has the effect of attenuating the input signal at high frequencies. Since the amplifier’s internal noise sources do not experience this attenuation, an impractically low signal-to-noise ratio is present at the amplifier’s output as a result.

#### 3.3.3. Nanopore Signal Dynamics

The CMOS interface design relies on the knowledge of the signal speed, as well. From an electronic signal processing perspective, the “speed” denotes the frequency bandwidth of the current signal that needs to be processed by CMOS amplification circuitry. Hence, the operational amplifier bandwidth needed by the ensuing CMOS circuitry is directly set by this aspect. Physically, the electronic current’s frequency profile is related to the rate at which the molecule of interest translocates through the nanopore. We now review the physical dynamics behind this rate.

The authors have noted the following temporal relationship for nanopores [65]:

(9) $$t_{P}=\frac{l}{N\langle v_{T}\rangle }\;[s/\text{base}]$$

where $t_{P}$ is the most probable translocation time [65], $\langle v_{T}\rangle$ is the “mean sliding velocity” [65], *l* is the physical length of DNA whose translocation is being considered and *N* is the number of nucleotides in that DNA. As already indicated above, $t_{P}$ relates the average time that it takes for a base to get through the pore.

The distribution of the amount of time it takes a base to translocate through a pore, that is the distribution of the “dwell time”, $t_{D}$, has been empirically expressed with [65]:

(10) $$P\left(t_{D}\right)=Ae^{-t_{D}/\tau_{1}}+Be^{-t_{D}/\tau_{2}}$$

where the time constants, $\tau_{1}$ and $\tau_{2}$, are sometimes referred to as the “short translocation time scale” and the “long translocation time scale”, respectively [65]. The long translocation time is related to the possible effects that the twists and turns of long molecule samples may have on the translocation time. Its effect becomes small for very short DNA samples (<50% of events achieve such time scales for N<3500). Ignoring the long translocation time scale, the average translocation time is simply $\tau_{1}$ for such a distribution. For state-of-the-art SSNs, measured dwell time values of around 0.5μs/nt have been reported [54] and, hence, average translocation rates of $v_{t}\approx 2.0\;\text{nt}/\mu s$ achieved. Such rates translate into electronic signal signatures with significant signal presence at high frequencies (i.e., >1 MHz). As alluded to above and discussed further in Section 5, this frequency can present a significant challenge for ensuing CMOS amplification stages and has historically been the motivation for efforts to slow the translocation of molecules through the pore [39,40,41]. Improvements in CMOS amplification can help alleviate the need for such efforts and help pave the way for solid-state nanopore adoption.

## 4. Nanopore-CMOS Interface

**Figure 11:**
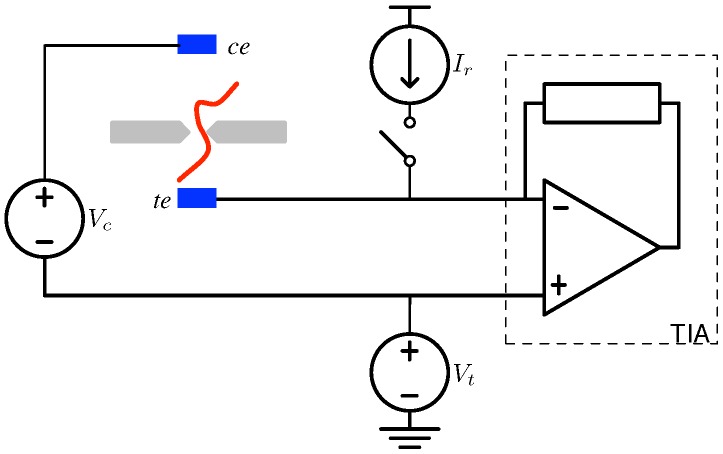
Basic electrical configuration employed in a nanopore test apparatus.

As outlined in general above, the basic nanopore arrangement consists of a pore-infused membrane between two electrodes. A functional representation of this is shown in Figure 11, which includes a transimpedance amplifier (TIA), along with bias controls $V_{c}$ and $V_{t}$, plus a reference current source $I_{r}$ included for calibration purposes. A discussion of the TIA and its role in efficient interfacing is presented in Section 5. In this section, we outline the physical means by which nanopores have been interfaced to their electronic components in the past and state-of-the-art techniques propelling these methods into the future.

In a research setting, the core biological nanopore test apparatus is typically fashioned from an inert material, such as Delrin plastic or Teflon, that is further housed in a metal block for mechanical support and protection and connected to a thermoelectric device for temperature regulation. Within the core polymer structure, which may measure roughly 5 cm on a side, are housed the *cis* and *trans* chambers. Fluidic and electrical connections are made to these chambers with silver/silver chloride (Ag/AgCl) contacts to realize a non-polarizable electrode-electrolyte interface.

**Figure 12:**
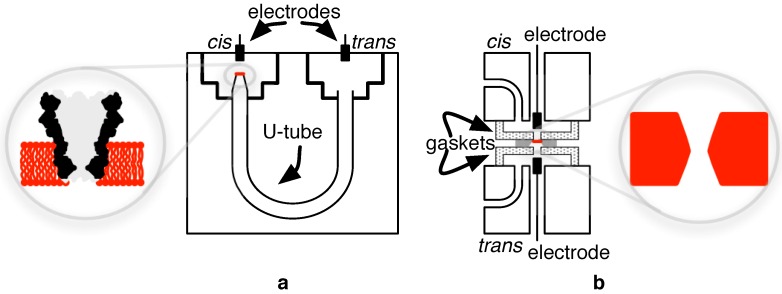
Examples of (**a**) a U-tube apparatus for biological nanopore testing and (**b**) its counterpart for solid-state nanopore testing.

A small aperture, around 20 μm in diameter, is machined between the two reservoirs, and it is within this opening that the lipid bilayer support membrane is typically made and, hence, used to support a nanopore molecule. A relatively intricate manual protocol is used to accomplish this [66,67], and the apparatus is specially designed (e.g., chambers arranged in the same horizontal plane and attached by a U-tube, as shown in Figure 12) [68], to allow the execution of this procedure under microscopic guidance with the help of micropositioners. To minimize noise and vibrations, tests are usually conducted within a Faraday box on an optical table.

Similar to their biological counterparts, experimental solid-state nanopore studies house their nano-device in a small non-reactive flow cell. In a typical set-up, the nanopore and its silicon membrane are tightly clamped between two gaskets which constitute the *cis* and *trans* chambers. The slight variations in procedure (i.e., the need for a tight clamp) compared to constructs meant for biological nanopore testing may require adjustments to materials used (e.g., chemical inertness, mechanical hardness, disposability, etc.) as addressed by the likes of PDMS, PEEK, PTFE, etc. Overall, the construction of the test apparatus is simplified for the solid-state pore relative to the biological pore.

The experimental arrangements outlined above, although well established as research methods, clearly preclude a close interface to CMOS technology. Instead, these procedures require a relatively bulky interconnect between the sensor and its electronic amplification components that introduce substantial electronic parasitics and, hence, performance degradation. Many researchers have responded to these problems indirectly by focusing on improving the sensor itself rather than its interface. Although this has resulted in critical benefits, means of directly addressing the problem by creating efficient, and arrayed, nanopore interfaces to custom microelectronic technologies are only now emerging.

### 4.1. Biological Nanopore-CMOS Interfaces

Perhaps surprisingly at first impression, given the big difference between their underlying make-up, biological nanopores, rather than their solid-state counterparts, have demonstrated a more efficient interface to CMOS. This is largely due to the amenability of this sensing modality to accommodate on-demand room-temperature, fluidics-based fabrication techniques, which makes them especially suitable as part of a post-CMOS-processing add-on step. This is in contrast to their solid-state versions, which rely on relatively inflexible manufacturing methods best served by a fully-integrated process from the bottom-up.

**Figure 13:**
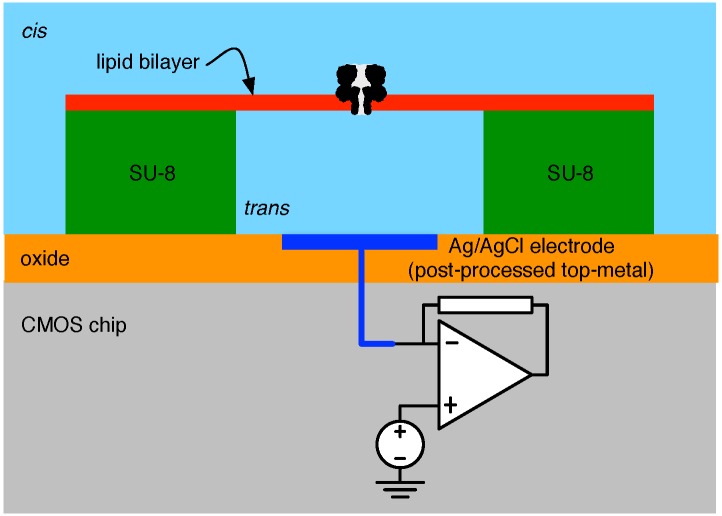
The cross-section of a biological nanopore-to-CMOS interface following the structure reported in [62].

A recent example of a close interface between CMOS and biological nanopores is given in [62], which employs the methods described in [69]. A cross-sectional representation of this structure is sketched in Figure 13. Herein, a *trans* chamber (micro-well) is fashioned using SU-8 photoresist over a silver electrode (to the input of a low-noise CMOS pre-amplifier) achieved by etching away a top-metal aluminium contact and, in its place, evaporating a 0.25 μm-thick Ag layer [70]. The SU-8 well is 20 μm in diameter and 5 μm thick, and its formation is followed by an electrode chloridisation. The well itself is then surrounded by a larger enclosure (not shown in Figure 13) ultimately intended to serve as the *cis* compartment. After filling the *trans* with electrolytic fluid, a manual bilayer formation technique similar to that outlined above is used.

Since the *trans* well formation is based on a simple lithographic process, the scaling of at least the structural aspect of this approach to an array of *trans* cells with a shared *cis* is obvious. Less clear is an automatic means of forming a support membrane and distributing nanopores therein in place of the manual methods largely employed to-date. Certain techniques have sought to scale and adapt the membrane formation methods briefly outlined above.

**Figure 14:**
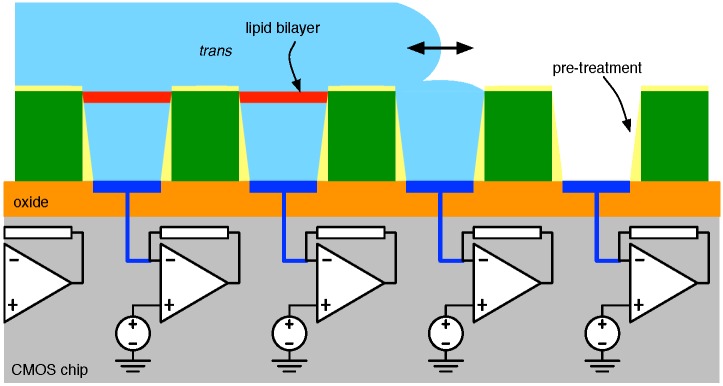
A micro-well array employed for automated membrane formation and pore insertion.

The mechanics of one such attempt [71] are illustrated in Figure 14. In particular, after *trans* chamber formation (e.g., using SU-8) using standard lithographic techniques atop a CMOS substrate, the empty micro-wells are subject to a pre-treatment with a hydrophobic medium (e.g., hexadecane in a non-polar solvent, such as hexane) intended to improve the adhesion of the amphiphilic molecules that make up the membrane to the well structures.

This is then followed by flowing an aqueous solution over the array, which, to start, fills the *trans* chambers. To enable the desired nanopore-based behaviour (i.e., modulated electrolyte current), the solution contains salt (e.g., KCl, NaCl, etc.), as well as buffer (e.g., PBS, HEPES, etc.) to maintain a desired level of pH. Critically, the solution also includes samples of amphipathic molecules of which the support membrane is comprised, phytanoyl lipids, such as DPhPC, are common for this purpose (although not ideal as noted below). Allowing the aqueous input to flow back-and-forth over a newly-filled well (e.g., Well 3 in Figure 14) achieves the formation of a bilayer membrane between the two media. Subsequent introduction of nanopore molecules into the flowing liquid results in the (stochastic) insertion of pores into the newly-formed membranes.

As alluded to above, the construction of nanopore membrane supports from naturally-occurring lipid molecules is not ideal. This is due to the relative fragility of such structures in the face of electrical stress and biological samples, two key elements of a nanopore-based sequencing technology. Instead, synthetic membranes are a much more robust option with silicone triblock copolymers, such as PMOXA-PDMS-PMOXAfinding particular preference [72]. However, the above membrane formation scheme has not proved amenable to the consistent formation of silicon triblock copolymer membranes. Instead, a means of arrayed nanopore interface formation based on static droplets [73] has been much more successful.

**Figure 15:**
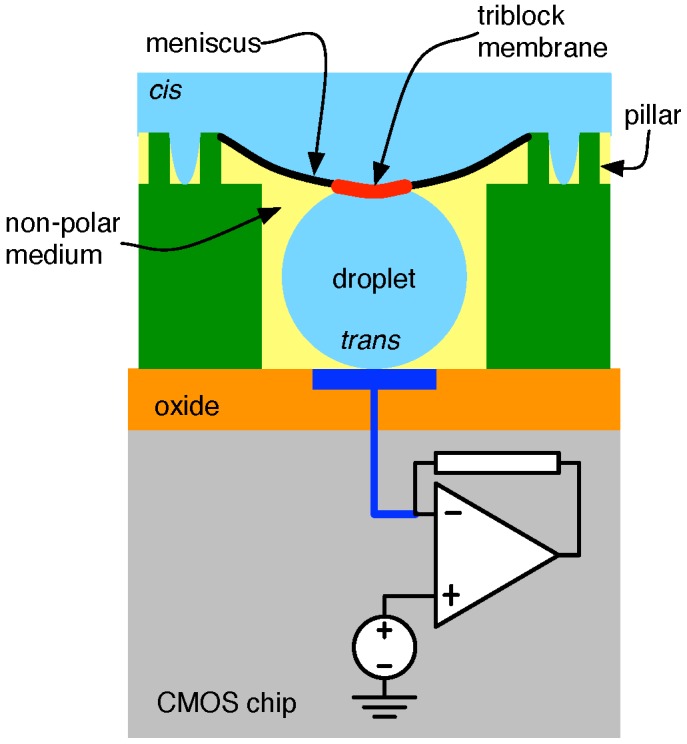
Cross-section of the micro-well with droplet formed *trans*, as described in [74].

In this scheme, arguably the state-of-the-art in nanopore-based sequencing interfaces, the *trans* chamber contents are made to consist of a polar droplet solution surrounded by a non-polar fluid (e.g., AR 20 silicone oil), as sketched in Figure 15 [74]. A convenient means of achieving this is by creating an hydrophilic/hydrophobic fluid emulsion along with a mixture of amphiphatic molecules [75]. Within such a mixture, droplets of polar material (e.g., KCl plus buffer) are formed with a surface coating of triblock. Droplet size can be controlled by the size of the apparatus used to mix the chemicals and their flow rates. Droplet diameters ranging between 5 and 500 μm are readily achievable, with diameters as small as 100-nm anticipated [75]. Adjacent droplets form bilayer membranes between one another, an outcome that can be extended to the formation of a layer between a droplet and a planar aqueous surface [76]. The amphiphatic molecules surrounding each droplet also stabilize them, preventing merging and, hence, a much more consistent droplet size.

An arrangement such as that shown in Figure 15 (showing one micro-well of an array) can be realized by constructing the droplet emulsion discussed above and allowing it to flow over a micro-well array built atop a CMOS chip. During this process, droplets are deposited in the like-sized wells, the rest of which is occupied by the non-polar medium in which they were emulsified. This is then followed by the flow of a triblock-infused secondary aqueous solution over the array to form the shared *cis* medium. The interaction between this secondary polar medium and the micro-well structure forms a meniscus, which connects with the *trans* droplet, allowing for the formation of a triblock membrane. A number of parameters influence the size of the interface, including the droplet size, the dimensions of the chamber recesses, as well as its ancillary features (e.g., surface patterns, intermediate pillar structures, etc.) [74]. The micro-well width is typically chosen to be 1.05–1.5× the droplet diameter, while their height (as well as that of the pillars) is chosen between 1.1–1.3× the droplet diameter.

**Figure 16:**
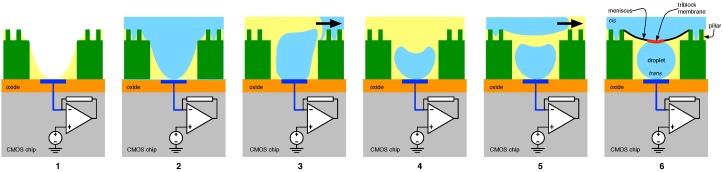
In situ formation of *trans* droplets in the micro-well array.

Perhaps even more advantageously, the *trans* droplet may be formed in situ in a method whose main steps are shown in Figure 16 [74]. In Step 1, a hydrophilic pre-treatment is applied to the array structure whose design is capable of wicking the fluid to its peripheries and away from the electrode (a potential problem with the method outlined above). In Step 2, the structure is flooded with aqueous solution (triblock infused), which fills all available recesses in the array. A subsequent flow of oil in Step 3 results in the formation of *cis* droplets (Step 4). An ensuing aqueous solution flow (Step 5) completes the aforementioned membrane interfaced *cis* and *trans* media (Step 6).

Ideally, all of the functionality needed to implement nanopore sequencing could be implemented on a single chip, but clearly technical and economic constraints preclude this option. As a result, connecting the CMOS chip most closely interfaced with the nanopore sensors (the “front-end” CMOS chip) to other system components (e.g., data converters, filters, digital signal processors, communications, etc.) becomes another critical consideration in nanopore-CMOS interfaces. The complexity of this problem grows as the number of sensors interfaced to the front-end grows. State-of-the-art designs from ONT achieve a 2048 micro-well array with a pitch of roughly 200-μm distributed over a footprint of roughly 6×15 ${\mathrm{mm}}^{2}$.

**Figure 17:**
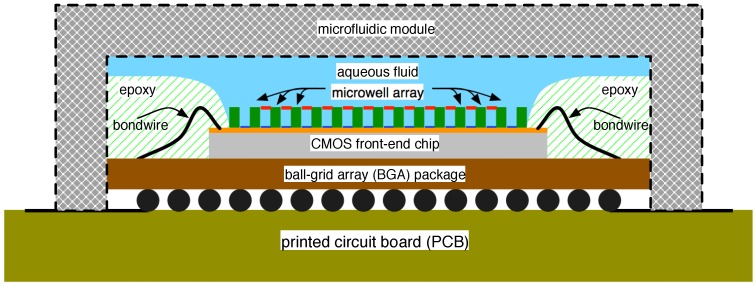
Cross-section of the example construct for connecting the nanopore sensor system to external system components.

A straightforward means of accommodating such a system could be carried out as shown in Figure 17. Herein, a CMOS front-end is affixed to a ball-grid array (BGA) package and connected to its output leads via bond wires. The package is bonded to a printed circuit board (PCB) to which other system components (not shown in Figure 17) are attached. Rather than covering the entire chip with an epoxy over-mould, only the bond wires may be covered, leaving the remainder of the chip exposed for the construction of the micro-well array. The complete apparatus can then be placed inside a specially-designed microfluidic module and any of the membrane formation and pore insertion steps outlined above applied.

Presently, only one in four micro-wells are processed by the front-end chip at any time, requiring 512 channels on the CMOS front-end. Handled directly, such a system would require 512 output pins, a manageable proposition for higher-end packages, but still a burden for any efforts at realizing compact system and board designs that will only become greater as micro-well array density grows. Clearly, a means of multiplexing data off-chip through a smaller number of pins is needed. Although the functional complexity of any such ancillary components should be relatively easy to manage in CMOS, they contribute to yet another problem complicating the design of Figure 17: temperature management. The heat produced by CMOS front-end chips operating immediately next to biological molecular samples can significantly compromise the quality of the data gathered. Again, this problem escalates as the complexity and size scale.

**Figure 18:**
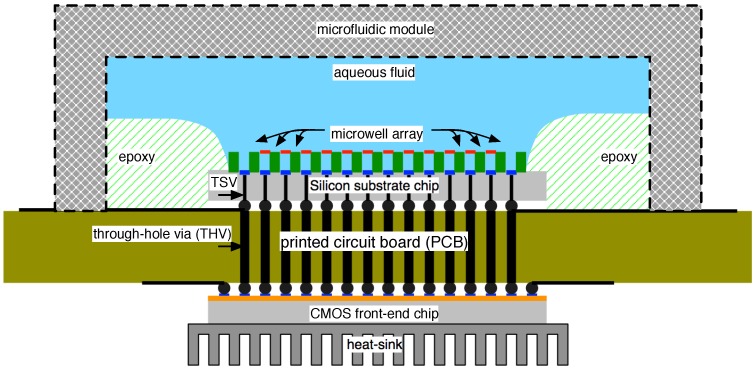
Cross-section of an example construct for connecting the nanopore sensor system to external system components. The construction of sensing structures and electronic structures on separate silicon substrates offers substantial room for the optimization of each component.

Many options are available to mitigate this, one of which is sketched in Figure 18. In this case, the purely electronic function is relegated to a CMOS chip separated from the sensor array by the PCB, which effectively servers as an interposer between the two (besides its role as a substrate for remaining signal processing and communications functions). A flip-chip on board (FCOB) bonding is assumed for the front-end [77]. This arrangement allows the front-end to retain a relatively close physical proximity to the sensors (hence controlling parasitic contributions) by through-hole vias (THVs), while allowing its direct interface to a heat-sink and, hence, the a means of thermal management.

The micro-well array in this example is built atop a separate silicon substrate (the “silicon sensor chip”) using standard lithographic techniques. A connection from the top of the silicon substrate to the bottom and, hence, the PCB is facilitated by the use of through-silicon vias (TSVs), an increasingly standard method in the realization of micro-electromechanical systems [78]. At production scales, TSVs can be made through substrates at least 350 μm thick, avoiding the need for wafer thinning, and can achieve via pitch less than 50 μm [79,80,81]. At the very least, micro-well array construction atop a separate substrate allows for the independent optimization of sensor, front-end and PCB designs. In the future, it is foreseeable that a growing number of functions (e.g., electronics for reference bias) could migrate to the silicon substrate chip, power permitting.

### 4.2. Solid-State Nanopore-CMOS Interfaces

Unlike their protein-based counterparts, nanopore-CMOS sequencing systems based on solid-state sensors are not yet commercially available. In fact, the same features that make solid-state sensors more robust in the face of external disturbances (mechanical vibrations, high voltages, biological reactions, etc.) complicate an efficient and economical interface to CMOS and, at least in part, delay their market availability. Whereas biologically-based pores (and associated membranes) can be fluidically formed again and again, thus enabling re-use, this option is not readily available to their solid-state counterparts. At the very least, this complicates system longevity for solid-state designs. Where biologically, systems could simply have their membranes chemically removed, *trans* chambers re-formed and electrode potentials replenished (e.g., by the inclusion of a suitable redox couple), followed by a reconstitution of pore and membrane, solid-state systems, lacking the ability for in situ reconstruction, would require some external fluidic access to their otherwise isolated chambers for the purpose of cleaning and replenishing their required media.

**Figure 19:**
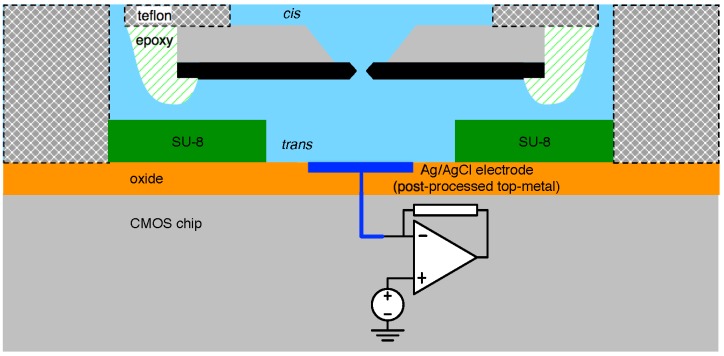
CMOS to solid-state nanopore interface as described in [82]. The nanopore chip is affixed to a non-reactive cell material on top.

Arguably the most prominent recent example of a solid-state nanopore apparatus is that presented in [82], a cross-section of which is shown in Figure 19; herein, a nanopore chip is glued to an inert material, which constitutes the boundary between the *cis* and *trans* reservoirs. The design achieves two top-side openings allowing for the introduction of fluid into the two chambers after pore construction. Excellent accuracy was achieved using this apparatus at very high translocation speeds, a result of minimal interconnect capacitance contributions due to the small separation between nanopore sensor and amplifying CMOS electronics.

**Figure 20:**
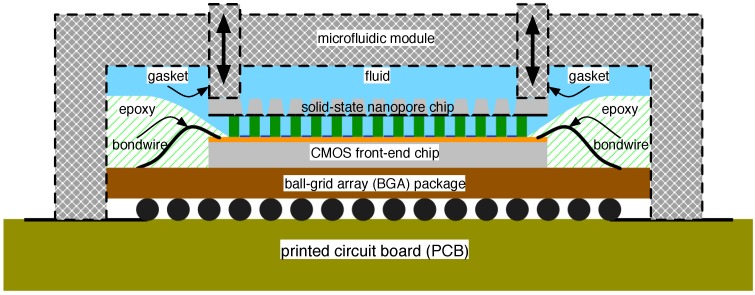
Cross-section of a solid-state nanopore array construct.

Scaling the above approach to an array of 1000 s of sensors as now found with biological pores is problematic for the aforementioned reason of filling the individual chambers while keeping them electrically isolated during test. Allowing for a mechanically-adjustable set-up, however, may be a suitable compromise. The cross-section of such a scheme is shown in Figure 20. Therein, the CMOS front-end chip is arranged as previously shown in Figure 17 regarding biological pore arrays. Unlike the biological scenario, however, a second chip hosting an array of solid-state nanopores is mechanically fixed to the CMOS front-end via movable gaskets. In such a set-up, the nanopore chip could start in the detached state, suspended above the CMOS chip. The appropriate fluid could then be introduced into the apparatus effectively filling the *cis* and *trans* chambers. Finally, the nanopore chip, affixed to the movable gaskets, could be manoeuvred down to make a tight physical connection with the CMOS chip, thus realizing isolated *trans* chambers, each coupled to the communal *cis* via a nanopore. This mechanism can be translated into the board-mediated approach sketched in Figure 18, as well.

**Figure 21:**
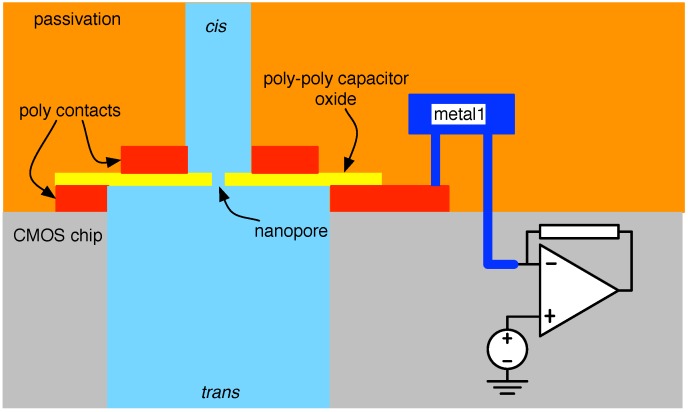
Direct implementation of a nanopore in an active CMOS substrate.

The logical conclusion to solid-state nanopore/CMOS interfaces would seem to be the direct implementation of the pores in active CMOS substrates; an interesting method of doing so is discussed in [83,84]. In particular, nanopores are created in the poly-insulator-poly (PIP) capacitors available in a mature 500-nm process, as illustrated in Figure 21. Herein, the PIP’s polysilicon terminals implement the *cis*/*trans* electrodes, and its 35-nm silicon-dioxide (${\mathrm{SiO}}_{2}$) insulator serves as the membrane within which the nanopore is ultimately etched.

The PIP is chosen as the nanopore support for its superior oxide quality. As noted in [84], the use of low-pressure chemical vapour deposition (LPCVD) as opposed to plasma-enhanced CVD leaves this oxide with less pinholes. Still, the low density of the ${\mathrm{SiO}}_{2}$ membrane was unable to prevent the substantial flow of $K^{+}$ ions through it. To prevent this permeability, the insulator was coated with ${\mathrm{Al}}_{2}O_{3}$ using a low-temperature atomic layer deposition process. Importantly, [84] showed that the substantial plasma treatments to which the CMOS wafer was subject did not noticeably effect the underlying circuit performance (of an on-chip DC current bias source).

## 5. Electronic Amplification

A sophisticated sequence of circuitry is needed to process the minute current signal (e.g., see Figure 5) available to the nanopore sensor. The three most prominent features of this sequence include signal amplification, filtering and sampling before core sequencing methods, like base calling and alignment, can be carried out in a digital computer. Of these three, the first, amplification, has arguably the greatest influence on the remaining signal processing. It is this component that ultimately sets the speed at which raw measured signals can be processed and the fidelity of the signal passed on to the remaining components.

**Figure 22:**
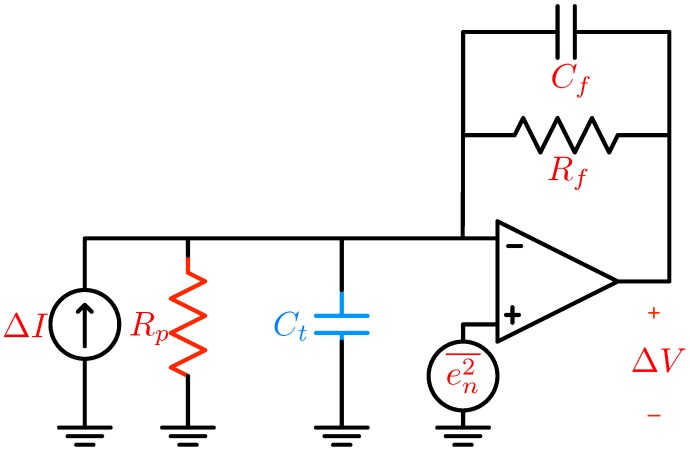
Basic transimpedance amplifier (TIA) implementation.

A simple representation of this amplification unit, the trans-impedance amplifier (TIA) [85], is shown in Figure 22. Assuming an ideal op-amp and negligible feedback capacitance $C_{f}$, the input/output relationship of this circuit is simply:

(11) $$\Delta V=R_{f}\Delta I$$

where, as discussed in Section 3, ΔI is the maximum current swing (i.e., modulation depth) possible for a given pore and ΔV is the corresponding voltage swing to which it is transformed. The purpose of this transformation is to simplify the job of signal sampling (typically done in the voltage domain) for the purpose of digitization. Typically, ΔV is limited by the DC supply voltage used to power the electronic circuits; modern CMOS technologies could thus accommodate ΔV≈1 V. For SSN currents of ΔI≈5 nA, a feedback resistance of $R_{f}\approx 200$ MΩ would be needed.

A direct implementation of such a large resistance on a CMOS chip is not practical. Resistance footprints are roughly 1-kΩ/${\mu m}^{2}$ and generally do not benefit from the scaling benefits (i.e., Moore’s law) driving transistor size reduction. Such space requirement could easily exceed the total active (i.e., transistor) silicon area requirement by an order of magnitude. Even more importantly, the unwanted (i.e., parasitic) capacitance associated with such a resistor would be tremendous (roughly 10 pF), therefore greatly increasing the effecting $C_{f}$ and, thus, limiting the speed handling capability of the circuit to frequencies below 100 Hz.

**Figure 23:**
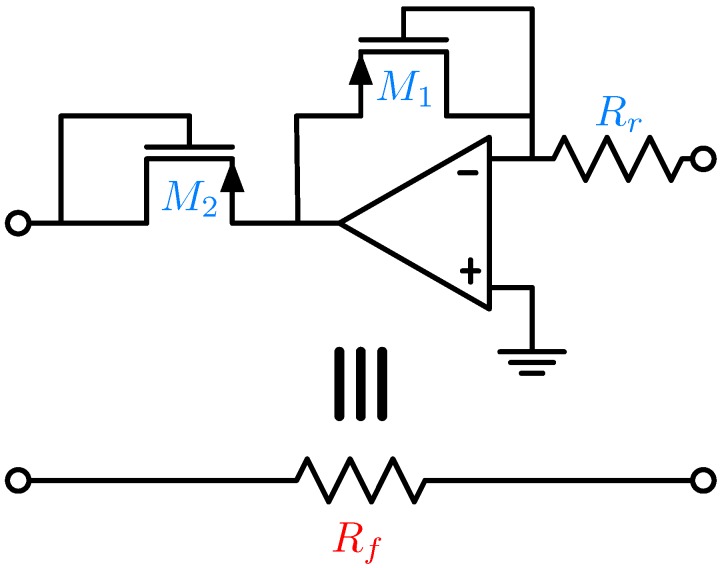
Active implementation of the feedback resistance, $R_{f}$.

Fortunately, CMOS affords the realization of an active (i.e., transistor-based), $R_{f}$, as demonstrated by the circuit shown in Figure 23 [86]. Herein, a reference resistor $R_{r}$ orders of magnitude smaller is used to establish a current, which undergoes a compressive current-to-voltage-to-current transformation via the transistors $M_{1}$ and $M_{2}$. As a result the current through the circuit is linearly attenuated depending on the ratio of the transistor sizes leading to the linear relationship $R_{f}=M\cdot R_{r}$, where *M* can easily be made in excess of 100.

The active implementation of $R_{f}$ is much smaller with negligible capacitive parasitics compared to its passive on-chip counterpart.

Alas, for nanopore applications, the active $R_{f}$ remedy is not sufficient. The combination of a large $R_{f}$ and a potentially large net membrane/interface capacitance $C_{t}$ conspires to compromise the feedback action of the amplifier and, thus, undermine its ability to implement the simple transformation (11) over sufficiently high frequencies. This is exactly, the effect that compromises the noise behaviour of the nanopore signal processing chain at high frequencies, an impact often referred to as “capacitive noise” or “dielectric noise” by the nanopore community. However, rather than being a direct noise boost, this is just the result of signal attenuation at high frequencies.

**Figure 24:**
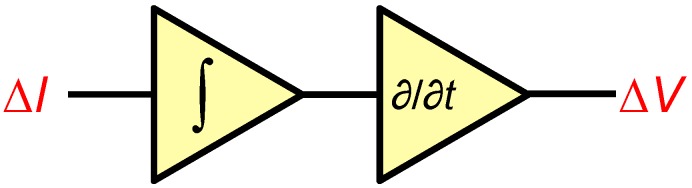
A two-stage integrator/differentiator amplification method for nanopore signals.

A classical fix to the above quandary (i.e., high $R_{f}$ for gain, but ensuing speed compromise in combination with $C_{t}$) [87] is to abandon attempts at achieving signal boosts in one stage. Rather, as shown in Figure 24, a gain with a low-pass (i.e., integrator-like), but controllable gain transfer function can be applied, followed by a high-pass (i.e., differentiator-like) gain transfer function. The combination of these results in the basic (i.e., constant with frequency) relationship 11 originally sought; the low-pass design of the first stage makes its performance much less susceptible to the vagaries of $R_{f}$ and $C_{t}$ while the high-pass stage, being freed from the influence of these components, can be reasonably designed for sufficient speed at the required frequencies.

**Figure 25:**
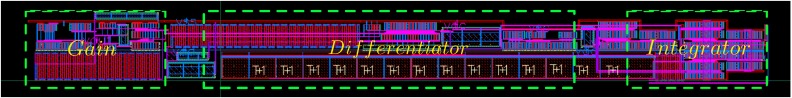
The layout of the integrator-differentiator transimpedance amplifier (TIA) in 130-nm CMOS.

A fully-integrated version of such a circuit in a 130-nm CMOS technology is described in [88]. Simulation results on an extracted layout of the circuit show its ability to achieve an effective $R_{f}$ of 200 MΩ up to a bandwidth of 4-MHz. Compared to recent integrated designs for high-speed low-current amplification in the same space [82,85], the reported design operates with a comparable noise while consuming roughly 10× less area and 3× less power. Specific performance comparisons are summarized in Table 1. A picture of the layout of this integrator-differentiator chain is shown in Figure 25. The implementation measures $40\times 400\;{\mu m}^{2}$.

## 6. Conclusions

Nanopores are a conceptually simple technology: nano-sized holes in a thin membrane. Molecules translocating the hole modulate an electrochemical current in a way proportional to that molecule’s underlying structure. The most prominent application of this method today is for the purpose of DNA sequencing; a use that can be carried out orders-of-magnitude more quickly per sensor than existing methods. These speeds, the possibility of increasing them and the opportunity to process nanopore signals with improved levels of accuracy in a miniaturized platform can only be achieved if nanopores are suitably interfaced to CMOS microelectronics, the most capable and efficient signal processing technology available. The planar nature of nanopore structures and their reliance on an electrochemical current modality makes these sensors well suited to such an interface.

In this paper, we reviewed the existing DNA methods, all of which rely on intricate chemical preparation and relatively slow chemical reactions to instigate sequencing signals. We then reviewed the state-of-the-art in nanopore sensors, the dimensions and construction of biological and solid-state manifestations. The signal characteristics of the pore were outlined and the sensor’s equivalent model detailed showing the potential for signal modulation depths on the order of 5-nA in solid-state pores, but also highlighting the deleterious presence of the membrane capacitance. Means of dealing with this problem in CMOS were also reviewed and outlined.

A variety of nanopore/CMOS interfaces were discussed. These included both multiple and single-chip solutions. Methods of automatically forming biological pore arrays interfaced to silicon, as well as solid-state pore arrays were shown.

## Figures and Tables

**Table 1:** Low-current CMOS amplifier comparison.

	This Work [88]	[82]	[85]
Technology	130-nm CMOS	130-nm CMOS	350-nm CMOS
Bandwidth	4 MHz	1 MHz	4 MHz
Supply	1.5 V	1.5 V	±1.5 V
Total power consumption	13 mW	N/A	40 mW
Integrator power consumption	2.3 mW	5 mW	N/A
Core area	0.016 mm^2^	0.2 mm^2^	0.34 mm^2^
Input voltage noise	4 nV/$\sqrt{\text{Hz}}$	5 nV/$\sqrt{\text{Hz}}$	3 nV/$\sqrt{\text{Hz}}$

## References

[1] Sanger F., Nicklen S., Coulson A. (1977). DNA sequencing with chain-terminating inhibitors. Proc. Natl. Acad. Sci. USA.

[2] Hartwell L.H., Hood L., Goldberg M.L., Reynolds A.E., Silver L.M. (2011). Genetics: From Genes to Genomes.

[3] Smith L.M., Sanders J.Z., Kaiser R.J., Hughes P., Dodd C., Connell C.R., Heiner C., Kent S.B.H., Hood L.E. (1986). Fluorescence detection in automated DNA sequence analysis. Nature.

[4] Marsh M., Tu O., Dolnik V., Roach D., Solomon N., Bechtol K., Smietana P., Wang L., Li X., Cartwright P. (1997). High-throughput DNA sequencing on a capillary array electrophoresis system. J. Capillary Electrophor..

[5] Loman N.J., Misra R.V., Dallman T.J., Constantinidou C., Gharbia S.E., Wain J., Pallen M.J. (2012). Performance comparison of benchtop high-throughput sequencing platforms. Nat. Biotech..

[6] Balasubramanian S. (1999). Polynucleotide Sequencing.

[7] Nyren P. (2001). Method of Sequencing DNA Based on the Detection of the Release of Pyrophosphate and Enzymatic Nucleotide Degradation.

[8] Margulies M., Egholm M., Altman W.E., Attiya S., Bader J.S., Bemben L.A., Berka J., Braverman M.S., Chen Y.-J., Chen Z. (2005). Genome Sequencing in Microfabricated High-Density Picolitre Reactors. Nature.

[9] Rothberg J.M., Hinz W., Rearick T.M., Schultz J., Mileski W., Davey M., Leamon J.H., Johnson K., Milgrew M.J., Edwards M. (2011). An Integrated Semiconductor Device Enabling Non-Optical Genome Sequencing. Nature.

[10] Mullis K.B. (1987). Process for Amplifying Nucleic Acid Sequences.

[11] Wanunu M., Cohen-Karni D., Johnson R.R., Fields L., Benner J., Peterman N., Zheng Y., Klein M.L., Drndic M. (2011). Discrimination of Methylcytosine from Hydroxymethylcytosine in DNA Molecules. J. Am. Chem. Soc..

[12] Shim J., Humphreys G.I., Venkatesan B.M., Munz J.M., Zou X., Sathe C., Schluten K., Kosari F., Nardulli A.M., Vasmatzis G. (2013). Detection and Quantification of Methylation in DNA using Solid-State Nanopores. Sci. Rep..

[13] Shim J., Kim Y., Humphreys G.I., Nardulli A.M., Kosari F., Vasmatzis G., Taylor W.R., Ahlquist D.A., Myong S., Bashir R. (2015). Nanopore-Based Assay for Detection of Methylation in Double-Stranded DNA Fragments. ACS Nano.

[14] Mardis E.R. (2013). Next-Generation Sequencing Platforms. Annu. Rev. Anal. Chem..

[15] Merriman B., Rothberg J.M. (2012). Progress in Ion Torrent Semiconductor Chip Based Sequencing. Electrophoresis.

[16] Morozova O., Marra M.A. (2008). Applications of Next-Generation Sequencing Technologies in Functional Genomics. Genomics.

[17] Rothberg J.M., Hinz W., Johnson K.L., Bustillo J. (2013). Methods and Apparatus for Measuring Analytes using Large Scale FET Arrays.

[18] Bergveld P. (1970). Development of an Ion-Sensitive Solid-State Device for Neurophysiological Measurements. IEEE Trans. Biomed. Eng..

[19] Bergveld P. (2003). Thirty Years of ISFETOLOGY. Sens. Actuator B Chem..

[20] Bausells J., Carrabina J., Errachid A., Merlos A. (1999). Ion-Sensitive Field-Effect Transistors Fabricated in a Commercial CMOS Technology. Sens. Actuator B Chem..

[21] Milgrew M.J., Cumming D.R.S., Hammond P.A. The Fabrication of Scalable Multi-Sensor Arrays using Standard CMOS Technology. Proceedings of the IEEE Custom Integrated Circuits Conference.

[22] Milgrew M.J., Hammond P.A., Cumming D.R.S. (2004). The Development of Scalable Sensor Arrays Using Standard CMOS Technology. Sens. Actuator B Chem..

[23] Eid J., Fehr A., Gray J., Luong K., Lyle J., Otto G., Peluso P., Rank D., Baybayan P., Bettmann B. (2009). Real-Time DNA Sequencing form Single Polymerase Molecules. Science.

[24] Lundquist P.M., Zhong C.F., Zhao P., Tomaney A.B., Peluso P.S., Dixon J., Bettman B., Lacroix Y., Kwo D.P., McCullough E. (2008). Parallel Confocal Detection of Single Molecules in Real-Time. Opt. Lett..

[25] Clarke J., Wu H.C., Jayasinghe L., Patel A., Reid S., Bayley H. (2009). Continuous Base Identification for Single-Molecule Nanopore DNA Sequencing. Nat. Nanotechnol..

[26] Ip C., Loose M., Tyson J., de Cesare M., Brown B., Jain M., Leggett R., Eccles D., Zalunin V., Urban J. (2015). MinION Analysis and Reference Consortium: Phase 1 data release and analysis. F1000Research.

[27] Coulter W.H. (1953). Means of Counting Particles Suspended in a Fluid.

[28] Church G., Deamer D.W., Branton D., Baldarelli R., Kasianowicz J. (1998). Characterization of Individual Polymer Molecules Based on Monomer-Interface Interactions.

[29] Baldarelli R., Branton D., Church G., Deamer D.W., Akeson M., Kasianowicz J. (2000). Characterization of Individual Polymer Molecules Based on Monomer-Interface Interactions.

[30] Denison T.J., Sauer A., Golovchenko J., Meller A., Brandin E., Branton D. (2015). Characterization of Individual Polymer Molecules Based on Monomer-Interface Interactions.

[31] Branton D., Deamer D.W., Marziali A., Bayley H., Benner S.A., Butler T., Di Ventra M., Garaj S., Hibbs A., Huang X. (2008). The Potential and Challenges of Nanopore Sequencing. Nat. Biotechnol..

[32] Venkatesan B.M., Bashir R. (2011). Nanopore Sensors for Nucleic Acid Analysis. Nat. Nanotechnol..

[33] Deamer D., Akeson M., Branton D. (2016). Three Decades of Nanopore Sequencing. Nat. Biotechnol..

[34] Song L.Z., Hobaugh M.R., Shustack C., Cheley S., Bayley H., Gouaux J.E. (1996). Structure of Staphylococcal Alpha-Hemolysin, a Heptameric Transmembrane Pore. Science.

[35] Clarke J., Jayasinghe L., Reid T., Bayley H. (2011). Base-Detecting Pore.

[36] Derrington I.M., Butler T.Z., Collins M.D., Manrao E., Pavlenok M., Niederweis M., Gundlach J.H. (2010). Nanopore DNA Sequencing with MspA. Proc. Natl. Acad. Sci..

[37] Butler T.Z., Pavlenok M., Derrington I.M., Niederweis M., Gundlach J.H. (2008). Single-Molecule DNA Detection with an Engineered MspA Protein Nanopore. Proc. Natl. Acad. Sci..

[38] Goyal P., Krasteva P.V., Gerven N.V., Gubellini F., Van den Broeck I., Troupiotis-Tsailaki A., Jonckheere W., Pehau-Arnaudet G., Pinkner J.S., Chapman M.R. (2014). Structural and Mechanistic Insights into the Bacterial Amyloid Secretion Channel CsgG. Nature.

[39] Manrao E.A., Derrington I.M., Laszlo A.H., Langford K.W., Hopper M.K., Gillgren N., Pavlenok M., Niederweis M., Gundlach J.H. (2012). Reading DNA at a Single-Nucleotide Resolution with a Mutant MspA nanopore and phi29 DNA Polymerase. Nat. Biotech..

[40] Lieberman K.R., Cherf G.M., Moody M.J., Olasagasti F., Kolodji Y., Akeson M. (2010). Progressive Replication of Single DNA Molecules in a Nanopore Catalyzed by phi29 DNA Polymerase. J. Am. Chem. Soc..

[41] Cherf G.M., Lieberman K.R., Rashid H., Lam C.E., Karplus K., Akeson M. (2012). Automated Forward and Reverse Ratcheting of DNA in a Nanopore at 5-ÅPrecision. Nat. Biotechnol..

[42] Ventra M.D., Taniguchi M. (2016). Decoding DNA, RNAand peptides with quantum tunnelling. Nat. Nanotechnol..

[43] Zwolak M., Ventra M.D. (2008). Physical Approaches to DNA Sequencing and Detection. Rev. Mod. Phys..

[44] Zwolak M., Ventra M.D. DNA Sequencing via Electron Tunnelling. Proceedings of the 2012 IEEE International Symposium on Circuits and Systems (ISCAS).

[45] Tsutsui M., Taniguchi M., Yokota K., Kawai T. (2010). Identifying Single Nucleotides by Tunnelling Current. Nat. Nanotechnol..

[46] Huang S., He J., Chang S., Zhang P., Liang F, Li S., Tuchband M., Fuhrmann A., Ros R., Lindsay S. (2010). Identifying Single Bases in a DNA Oligomer with Electron Tunnelling. Nat. Nanotechnol..

[47] Ivanov A.P., Freedman K.J., Kim M.J., Albrecht T., Edel J.B. (2014). High Precision Fabrication and Positioning of Nanoelectrodes in a Nanopore. ACS Nano.

[48] Heng J.B., Aksimentiev A., Ho C., Dimitrov V., Sorsch T.W., Miner J.F., Mansfield W.M., Schulten K., Timp G. (2005). Beyond the Gene Chip. Bell Labs Tech. J..

[49] Sigalov G., Comer J., Timp G., Aksimentiev A. (2008). Detection of DNA Sequences Using and Alternating Electric Field in a Nanopore Capacitor. Nano Lett..

[50] Gracheva M.E., Xiong A., Aksimentiev A., Schulten K., Timp G., Leburton J.P. (2006). Simulation of the Electric Response of DNA Translocation Through a Semiconductor Nanopore-Capacitor. Nanotechnology.

[51] Leroux A., Destine J., Vanderheyden B., Gracheva M.E., Leburton J.P. (2010). SPICE Circuit Simulation of the Electrical Response of a Semiconductor Membrane to a Single-Stranded DNA Translocating Through a Nanopore. IEEE Trans. Nanotech..

[52] Li J., Stein D., McMullan C., Branton D., Aziz M.J., Golovchenko J.A. (2001). Ion-Beam Sculpting at Nanometre Length Scales. Nature.

[53] Heerema S.J., Dekker C. (2016). Graphene Nanodevices for DNA Sequencing. Nat. Nanotechnol..

[54] Venta K., Shemer G., Puster M., Rodriguez-Manzo J.A., Balan A., Rosenstein J.K., Shepard K., Drndic M. (2013). Differentiation of Short, Single-Stranded DNA Homopolymers in Solid-State Nanopores. ACS Nano.

[55] Rodriguez-Manzo J.A., Puster M., Nicolai A., Meunier V., Drndic M. (2015). DNA Translocation in Nanometer Thick Silicon Nanopores. ACS Nano.

[56] Dimitrov V., Mirsaidov U., Wang D., Sorsch T., Mansfield W., Miner J., Klemens F., Cirelli R., Yemenicioglu S., Timp G. (2010). Nanopores in Solid-State Membranes Engineered for Single-Molecule Detection. Nanotechnology.

[57] Wang J., Ma J., Ni Z., Zhang L., Hu G. (2014). Effects of Access Resistance on the Resistive-pulse Caused by Translocating of a Nanoparticle through a Nanopore. RCS Adv..

[58] Carlsen A.T., Zahid O.K., Ruzicka J., Taylor E.W., Hall A.R. (2014). Interpreting the Conductance Blockades of DNA Translocations through Solid-State Nanopores. ACS Nano.

[59] Kowalczyk S.W., Grosberg A.Y., Rabin Y., Dekker C. (2011). Modeling the Conductance and DNA Blockade of Solid-State Nanopores. Nanotechnology.

[60] Willmott G.R., Smith B.G. (2012). Comment on ‘Modeling the Conductance and DNA Blockade of Solid-State Nanopores’. Nanotechnology.

[61] Kowalczyk S.W., Grosberg A.Y., Rabin Y., Dekker C. (2012). Reply to Comment on ‘Modeling the Conductance and DNA Blockade of Solid-State Nanopores’. Nanotechnology.

[62] Rosenstein J.K., Ramakrishnan S., Roseman J., Shepard K.L. (2013). Single Ion Channel Recordings with CMOS-Anchored Lipid Membranes. Nano Lett..

[63] Balan A., Machielse B., Niedzwiecki D., Lin J., Ong P., Engelke R., Shepard K.L., Drndic M. (2014). Improving Signal-to-Noise Performance for DNA Translocation in Solid-State Nanopores at MHz Bandwidths. Nano Lett..

[64] Smeets R.M.M., Keyser U.F., Dekker N.H., Dekker C. (2008). Noise in Solid-State Nanopores. Proc. Natl. Acad. Sci. USA.

[65] Wanunu M., Sutin J., McNally B., Chow A., Meller A. (2008). DNA Translocation Governed by Interactions with Solid-State Nanopores. Biophys. J..

[66] Montal M., Mueller P. (1972). Formation of Bimolecular Membranes from Lipid Monolayers and a Study of Their Electrical Properties. Proc. Natl. Acad. Sci. USA.

[67] Gutsmann T., Heimburg T., Keyser U., Mahendran K.R., Winterhalter M. (2015). Protein Reconstitution Into Freestanding Planar Lipid Membranes for Electrophysiological Characterization. Nat. Protoc..

[68] Akeson M., Branton D., Kasianowicz J.J., Brandin E., Deamer D.W. (1999). Microsecond Time-Scale Discrimination Among Polycytidylic Acid, Polyadenylic Acid, and Polyuridylic Acid as Homopolymers or as Segments Within Single RNA Molecules. Biophys. J..

[69] Baaken G., Sondermann M., Schlemmer C., Ruhe J., Behrends J.C. (2008). Planar microelectrode-cavity array for high-resolution and parallel electrical recording of membrane ionic currents. Lab Chip.

[70] Polk B.J., Stelzenmuller A., Mijares G., MacCrehan W., Gaitan M. (2006). Ag/AgCl Microelectrodes with Improved Stability for Microfluidics. Sens. Actuators B Chem..

[71] Reid S.W., Reid T.A., Clarke J.A., White S.P., Sanghera G.S. (2009). Formation of Layers of Amphiphilic Molecules.

[72] Gonzalez-Perez A., Stibius K.B., Vissing T., Nielsen C.H., Mouritsen O.G. (2009). Biomimetic Triblock Copolymer Membrane Arrays: A Stable Template for Functional Membrane Proteins. Langmuir.

[73] Sun M., Bithi S.S., Vanapalli S.A. (2011). Microfluidic Static Droplet Arrays with Tuneable Gradients in Material Composition. Lab Chip..

[74] Hyde J.R., Bahamon P.M.O., Brown C.G., Heron A.J., Mackett P.R. (2015). Formation of Array of Membranes and Apparatus Therefor.

[75] Bayley J.H.P., Holden M., Heron A.J., Needham D. (2012). Formation of Bilayers of Amphipathic Molecules.

[76] Wallace M.I., Heron A.J., Holden M.A. (2007). Bilayers.

[77] Laub J.H. (2000). Low Cost Flip Chip Technologies for DCA, WLCSP, and PBGA Assemblies.

[78] Ebefors T., Fredlund J., Perttu D., van Dijk R., Cifola L., Kaunisto M., Rantakari P., Vähä-Heikkilä T. The Development and Evaluation of RF TSV for 3D IPD Applications. Proceedings of the 2013 IEEE International 3D Systems Integration Conference (3DIC).

[79] Kalvesten E., Ebefors T., Svedin N., Rangsten P., Schonberg T. (2003). Electrical Connections in Substrates.

[80] Bauer T. (2011). First High Volume Via Process for Packaging and Integration of MEMS/CMOS.

[81] Kalvesten E., Ebefors T., Svedin N., Eriksson A. (2013). Bonding Process and Bonded Structures.

[82] Rosenstein J.K., Wanunu M., Merchant C.A., Drndic M., Shepard K.L. (2012). Integrated nanopore sensing platform with sub-microsecond temporal resolution. Nat. Methods.

[83] Uddin A., Yemenicioglu S., Chen C.H., Corgliano E., Milaninia K., Xia F., Plaxco K., Theogarajan L. Biosensing with Integrated CMOS Nanopores. Proceedings of the SPIE.

[84] Uddin A., Yemenicioglu S., Chen C.H., Corgliano E., Milaninia K., Theogarajan L. (2013). Integration of Solid-State Nanopores in a 0.5 μm CMOS Foundry Process. Nanotechnology.

[85] Ferrari G., Gozzini F., Molari A., Sampietro M. (2009). Transimpedance Amplifier for High Sensitivity Current Measurements on Nanodevices. IEEE J. Solid-State Circuits.

[86] Kim J., Maitra R., Pedrotti K.D., Dunbar W.B. (2013). A Patch-Clamp ASIC for Nanopore-Based DNA Analysis. IEEE Trans. Biomed. Circuits Syst..

[87] Sigworth F.J., Sakmann B., Neher E. (1995). Electronic Design of the Patch Clamp. Single-Channel Recording.

[88] Huang Y., Magierowski S., Ghafar-Zadeh E. (2016). CMOS for High-Speed Nanopore DNA Basecalling. http://waset.org/publications/10001103/cmos-solid-statenanopore-dna-system-level-sequencing-techniques-enhancement.

